# Supplementary material for: Chromothripsis during telomere crisis is independent of NHEJ, and consistent with a replicative origin
Source: Genome Res. 2019 May;29(5):737–49. doi: 10.1101/gr.240705.118 (PMC6499312; doi:10.1101/gr.240705.118)
Supplement: Supplemental Material [file supp_gr.240705.118_Supplemental_file_1.zip › contigs/annotated_contigs/DB46/contig.2.DB46_length_351_mean_cov_5.82336182336.docx]

**DB46_length_351_mean_cov_5.82336182336**

```
TGTATTGTTAATCACTTTTTTGGTCCCAAGAATAATATAATCACACAATCTCCTACTGTAGCCTTTTCTGTGTCCACTTTCTTTTCTCA
 >chr20:13411646-13411761 - E=8e-58
AAATTGTAAATAATGTTCTCATT|ACA|TTAGCCTATGTTCGCCAGGTTTTAGTAGATAAGCTTCATAAGCATCAACGCCTCCAGTTAA
                        >chr20:13413210-13413449 + E=1e-132
TGAACGGCAAGGTTTCACTTATTTCATAAACTGGCACTTCACACTTAATGCTTCCTCTTGAAATAAAGAGTAGGAACCAGTTTATAAAT

CACTACATTGGTAATAAAAACTATATAACGTGAGCTAAAATACTATGACTTATCCTTAAATATGCATTTCTGTACCACCAAAAATG
```
